# Circadian Profile of Salivary Melatonin Secretion in Hypoxic Ischemic Encephalopathy

**DOI:** 10.1155/2020/6209841

**Published:** 2020-09-25

**Authors:** Łukasz Kapek, Justyna Paprocka, Marek Kijonka, Maria Zych, Ewa Emich-Widera, Beata Rzepka-Migut, Damian Borys, Ilona Kaczmarczyk-Sedlak, Maria Sokół

**Affiliations:** ^1^Department of Medical Physics, Maria Skłodowska-Curie National Research Institute of Oncology, Gliwice, Poland; ^2^Faculty of Science and Technology, University of Silesia, Katowice, Poland; ^3^Department of Paediatric Neurology, Faculty of Medical Sciences in Katowice, Medical University of Silesia, Katowice, Poland; ^4^Department of Pharmacognosy and Phytochemistry, Faculty of Pharmaceutical Sciences in Sosnowiec, Medical University of Silesia, Katowice, Poland; ^5^Department of Pediatric Neurology, St. Queen Jadwiga's Regional Clinical Hospital No. 2, Rzeszów, Poland; ^6^Silesian University of Technology, Department of Systems Biology and Engineering, Gliwice, Poland

## Abstract

**Purpose:**

In the present study, the salivary melatonin secretion in the hypoxic ischemic encephalopathy (HIE) children was measured. The logit model was fitted to the data to obtain the salivary dim light melatonin onsets (DLMOs), and the results were compared with the values estimated from the classic threshold method with a linear interpolation and those previously published for the blood measurements.

**Materials and Methods:**

9 patients suffering from HIE aged from 65 to 80 months were included in the study. The melatonin levels were assessed by a radioimmunoassay (RIA). The diurnal melatonin secretion was estimated using a nonlinear least squares method. Student's *t*-test and the Mann–Whitney *U* test were used for the comparisons of the obtained parameters.

**Results:**

The circadian profiles of the melatonin secretion for both calculation methods do not differ statistically. The DLMO parameters obtained in the blood and saliva samples in children with hypoxic ischemic encephalopathy were similar.

## 1. Introduction

Melatonin (N-acetyl-5-methoxytryptamine), secreted mainly by the pineal gland, but synthesized also in many other tissues and cells, diffuses into blood plasma and saliva, where it can be measured experimentally. Because it is involved in the regulation of circadian rhythms, such as the sleep-wake rhythm, neuroendocrine rhythms, or body temperature cycles, the disturbances of its secretion are considered an early indicator of certain disorders and also the biomarker of their follow-up [1]. The most commonly reported phase marker of the melatonin secretion is the DLMO—the dim light melatonin onset [2]. In physiological condition, it is particularly convenient, since it can usually be obtained about 2 to 3 hours prior to a habitual sleep.

In our previous studies, we showed that melatonin secretion in children with hypoxic ischemic encephalopathy (HIE) is significantly disturbed, even stronger than in epilepsy. Its characteristic features, as seen from the blood sampling [3, 4], are the delayed melatonin phase release and the shift of the DLMO parameters to the later morning hours.

The mathematical modelling of the circadian melatonin cycle was used by us to objectify the description of the melatonin secretion in blood [5]. Although many mathematical models are used to obtain the information on the circadian phase from the plasma melatonin rhythm [6], they may be insufficient when modelling the salivary melatonin secretion due to much lower concentration of melatonin in saliva than in the plasma. Moreover, because taking the saliva samples without disturbing a sleeping individual is difficult, the estimates may be of lower resolution, and in consequence, the lack of data from the entire time of melatonin secretion makes it impossible to use any threshold calculation that depends on the overall amplitude of the pulse [7].

Most of the modelling methods depend on curve-fitting of the melatonin profile and/or the crossing of a threshold to determine phase. The simple threshold interpolation is one of the most widely used for the DLMO description in endogenous melatonin secretion [8, 9], though it is claimed to be less accurate than the more flexible curve-fitting models [10, 11].

In saliva, the phase estimates are usually calculated by the same curve-fitting and threshold methods as for plasma melatonin, but the nonlinear shape of melatonin secretion and missing data may lead to the contradictory results. Therefore, more complex interpolations, biophysical models fitting, or the differential equation methods were introduced in the circadian rhythm analysis [4, 10, 12].

In our previous work [5], we applied a bell-shaped function to model the melatonin secretion in blood in the HIE children—such modelling requires a complete melatonin curve to calculate the DLMOs. The logit model proposed in this work makes it possible to estimate the DLMO parameters with a shorter melatonin collection time.

We used such model to fit to the melatonin salivary secretion onsets in the HIE children, and its results were compared with those obtained from the classic threshold method with linear interpolation. Then, the estimates of the salivary DLMO parameters were compared with the blood values from the previous study [5].

## 2. Materials and Methods

The study was approved by the Ethic Committee of the Medical University of Silesia in Katowice. The informed written consent was taken from the parent or caregivers. The study was carried out at the Department of Pediatric Neurology, School of Medicine in Katowice, the Medical University of Silesia in Katowice.

### 2.1. Patients

9 patients suffering from hypoxic ischemic encephalopathy aged from 61 to 82 months (mean age 5.92 years, SD ± 0.56) were included in the study from the group of 19 patients. 10 patients were rejected at the initial stage of the data analysis due to the missing data from the rising part of the salivary melatonin onset—in such cases, the phase markers could not be calculated.

The demographic characteristics of the participants are shown in Table 1. The recruitment procedure of the study group was the same as described in our previous work [5].

Before and during the experimental period, the subjects were not administered the medications affecting melatonin secretion, such as benzodiazepines and their antagonists, fluvoxamine, caffeine, vitamin B12, and nonsteroidal anti-inflammatory drugs (aspirin, ibuprofen, indomethacin, adrenolytics, prostaglandins inhibitors, calcium channel blockers, dexamethasone, and antidepressants). Furthermore, the legal guardians of the patients were instructed not to use a toothpaste or mouthwash during the assessments.

In 3 patients, the epileptic seizures did not occur on the day or the day before the melatonin measurements, and 6 children had them on the day of sampling. The amplitudes of the melatonin release were estimated for these patients using a nonparametric Mann–Whitney *U* test, and no statistical significance was found (*p*=0.3662).

### 2.2. Experimental Design

In order to determine the melatonin concentration and its circadian excretion profile, all subjects had their saliva taken every hour starting from 17:00 till 7:00 am. Saliva was collected in dim red light (10 lux) into the Salivette tubes (Sarstedt, Germany) by chewing on a cotton swab for 1-2 min. The sampling took place during the hospitalization at the Department of Pediatric Neurology, Medical University of Silesia in Katowice. During the collection, the patients stayed in a darkroom. The use of tablets and cells was prohibited.

The collected samples were shipped in dry ice to the laboratory to be radioimmunoassayed for melatonin detection. The experiment was performed under a dim light condition. The enzyme-linked immunosorbent assay (ELISA) method was used. The lower limit of sensitivity was determined by interpolating the mean optical density minus 2 SDs of 30 sets of duplicates at the 0 pg/mL level. The minimal concentration of melatonin that can be distinguished from 0 was 1.37 pg/mL. The functional sensitivity of the assay (Salivary Melatonin EIA Salimetrics) was 1.42 pg/ml, and the intra- and interassay coefficients of the variabilities were 0.2% and 16.6%, respectively.

### 2.3. Data Analysis

The circadian timing was determined by calculating the DLMO values as the marker of the individual circadian clock and in particular as an indicator of the beginning of the internal biological night. Moreover, the minimum and maximum melatonin concentrations were also calculated [8, 11, 13].

#### 2.3.1. Melatonin Secretion Parameters: Curve-Fitting Method

In order to describe the nonlinear character of the melatonin onset, the three parameter logit estimation was applied to the data [4, 10]. Due to the changes in the melatonin secretion amplitudes in pediatric patients, the relative thresholds were used in the melatonin profiles description to normalize the amplitude differences and facilitate the comparisons [2, 10].

The model is based on a time-dependent melatonin function MLT(t):

(1) $$\begin{array}{c} \text{MLT}\left(t\right)=\left(\frac{b_{1}}{\left(1+\exp\left(-t+b_{2}\right)\right)}\right)+b_{3}. \end{array}$$

The melatonin secretion parameters were normalized, and their biophysical interpretation is as follows:

1. *b*_1_ is a melatonin release amplitude (pg/mL), where the amplitude is the difference between the minimum and maximum melatonin concentrations
2. *b*_2_ (DLMO50E) denotes the time at which a melatonin level exceeds 50% amplitude (h), and *E* indicates the method of determining the parameter (E-estimation)
3. *b*_3 is a_ minimum melatonin concentration (pg/mL) from a range <0, +∞)
4. The maximum melatonin concentration *b*_max_ is a sum of *b*_1_ and *b*_3_

The dim light melatonin onset 25% (the time at which a melatonin level exceeds 25% amplitude) was calculated using equation (1):

(2) $$\begin{array}{c} {\text{DLMO}25}_{E}=b_{2}-\ln\left(3\right). \end{array}$$

**Figure 1:**
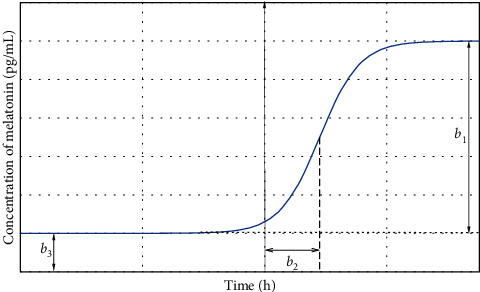
The melatonin secretion model. The solid line shows the curve-shaped function approximating the melatonin secretion with the following parameters: *b*_1_ is the melatonin release amplitude; *b*_2_ is DLMO_50E_; *b*_3_ is the minimum melatonin concentration; and dotted line is the minimum melatonin concentration level.

The phase and amplitude parameters may have different meanings in some situations depending on the used methods in secretion profile analysis [4, 5, 10, 14–17]. Thus, Figure 1 presents the graphical representation of the melatonin secretion model and visualizes the biophysical meaning of its parameters.

This set of parameters was estimated in order to define a melatonin cycle in saliva. The data were fitted with a nonlinear least squares fitting analysis based upon the Levenberg–Marquardt method in Statistica 12 software.

The quality of the obtained models was verified by the normality test of the residuals' distribution, the statistical significances of the estimated parameters, the percentage of the explained variance (>80%), and the *R* value (>0.89).

#### 2.3.2. Melatonin Secretion Parameters: Threshold-Based Method with Linear Interpolation

The salivary DLMO was also determined using the threshold-based methods that depend on the crossing of a predetermined melatonin concentration. A linear interpolation was used to determine the threshold-crossing time. The relative threshold of the 25th and 50th percentiles of the melatonin amplitude were chosen to obtain DLMO25I and DLMO50I, respectively (the I index denotes that the parameters were obtained via interpolation).

The minimum and maximum melatonin concentrations correspond to the lowest and highest values in the measured secretion profile, respectively.

#### 2.3.3. Statistical Comparison of the Melatonin Secretion

The obtained melatonin parameters (the minimum and maximum melatonin concentrations and the DLMO parameters) were interpreted in terms of their biophysical and clinical meaning and analyzed using Statistica 12 software. In order to compare the estimated (a curve-fitting method) secretion parameters with those obtained with the threshold-based method (with a linear interpolation), Student's *t*-test was used. Since, for the minimum melatonin concentration, the individual groups do not meet the requirements for the parametric tests (the data were not normally distributed), a nonparametric Mann–Whitney *U* test was used.

Finally, the salivary melatonin DLMO parameters estimated using the curve-fitting method were compared with those obtained from the blood samples of the HIE patients [5].

The values less than 0.05—a predetermined significance level—were accepted as indicating that the observed result would be highly unlikely under the null hypothesis.

## 3. Results

**Figure 2:**
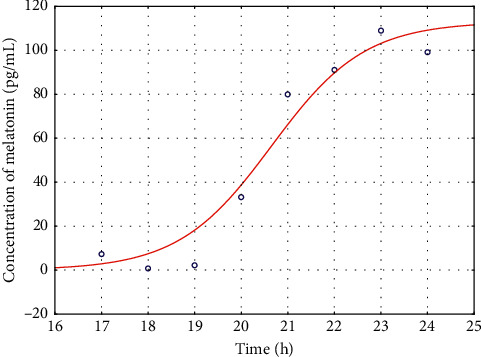
The salivary melatonin onset for the exemplary patient with hypoxic ischemic encephalopathy—the estimated model; and *ο* are the measured values.

The salivary melatonin onset curves were approximated for each patient separately, and the illustrative example of the estimated model obtained for a representative patient with hypoxic ischemic encephalopathy is shown in Figure 2.

The approximated parameters are *b*_1_ = 60,07 (pg/mL); *b*_2_ = 24.65 (h); *b*_3_ = 4.05 (pg/mL); *b*_max_ = 64.12 (pg/mL); and DLMO25 = 23.26 (h).

### 3.1. Comparison of the Salivary Melatonin Secretion Parameters Calculated from the Curve-Fitting and Threshold-Based (with a Linear Interpolation) Methods

The salivary melatonin secretion profiles (DLMO50, DLMO25, and maximum and minimum melatonin concentrations) estimated by the curve-fitting method were compared with that obtained using the traditional threshold-based method with a linear interpolation. The statistical analysis was performed using Student's *t*-test, and the results are presented in Tables 2 and 3. Moreover, the minimum melatonin concentrations were compared using Mann–Whitney–Wilcoxon because the requirements for the parametric tests were not fulfilled for this variable (the data were limited and not normally distributed).

### 3.2. Comparison of the Salivary and Blood Melatonin DLMO Parameters in Hypoxic Ischemic Encephalopathy

The salivary DLMO parameters obtained using a curve-fitting method were compared with those obtained from the blood secretion profiles also for the HIE group from our previous study [5] (Table 4). The results show more evidence for the null hypothesis of no difference of the salivary and blood DLMOs in the HIE groups. The statistical analysis of the other concentration parameters was not performed due to the expected significant disproportion of the melatonin concentrations values in both biofluids.

## 4. Discussion

Determination of the melatonin levels in saliva is the most popular method, due to its ease, relatively low invasiveness, and a relationship between the circadian changes of the melatonin concentration in saliva and the melatonin variations in plasma [18] or serum [19–21]. Also, the urinary excretion of 6-sulphatoxymelatonin (aMT6s), the major melatonin metabolite in humans, is proved to oscillate consistently with melatonin concentration in plasma and saliva [2, 22].

As we found in the PubMed database, in the last decade, the salivary melatonin measurements were mentioned in about 57% of the papers concerning the melatonin secretion, whereas about 25% related to its evaluation in blood or serum and about 18% in urine. The concentration of melatonin in saliva is 24–33% of the plasma melatonin (this percentage reflects the free melatonin fraction, not related to albumin), making its routine determination an analytical challenge [18, 23, 24]. Due to the volume differences of plasma and saliva, the interindividual differences in sensitivity to the light, a diurnal variation in melatonin synthesis, and the effects associated with the continuous production of saliva and low salivary melatonin concentrations, the salivary melatonin sampling is of lower resolution and sensitivity than in case of blood [2, 25]. Moreover, the salivary measurements are associated with the limitations resulting from eating, drinking, and oral hygiene measures that could falsify the results.

Thus, taking into account the evident quantitative advantage of the salivary measurements over those in other biofluids, but at the much lower accuracy of the salivary melatonin profile estimates, one may ask whether the latter are reliable and what is the actual correspondence of the results obtained for various biofluids. Though such comparisons are available for healthy subjects [26], there is a sparse number of the papers on the correlation between the salivary and blood melatonin levels in HIE children [2, 6, 27]. In our study, the salivary DLMO parameters obtained for the HIE children were compared with the blood phase markers of the children with the same diagnosis [5]. As revealed from the comparison, the DLMO values are consistent showing that the circadian melatonin phase markers in blood and saliva for the children with hypoxic ischemic encephalopathy are similar. Additionally, for both data sets, the curve-fitting method was applied in the DLMO calculations. Unfortunately, the differences in the data collection procedures did not allow to use one circadian model. However, when using different fitting curves in the estimation process, one must be aware that it may result in the uncertainties of the determined values. On the other hand, due to the frequent limitations of the saliva and blood collection methods, one universal melatonin secretion modelling method seems to be unavailable. Therefore, to facilitate the comparisons of the results and to verify them, some comparative testing should be applied [2], and in our study, the comparison of the salivary parameters with the blood melatonin ones plays such role. Though the saliva and blood samples were collected for two separate HIE groups, the inclusion criteria for the children with the same clinical diagnosis were the same.

In the estimations, we focused on the DLMO, as the most accepted and reliable circadian phase marker, claimed to be more reliable than DLMOoff (the dim light melatonin offset) and the phase markers derived from the core body temperature rhythm [11, 15]. On the other hand, the most diversity in the published methods occurs with determination of the onset of melatonin secretion. The main hypothesis of our work was that the DLMO parameters obtained via the estimation and interpolation methods are compatible. Moreover, since the full salivary melatonin profiles without missing data are usually difficult to be obtained, we tested the ability of the simplified model to predict the rising part of the melatonin synthesis onset accurately. Two methods of the DLMO determination from the salivary melatonin measurements in the HIE patients were compared: the threshold-based interpolation and the curve-fitting method. The logit model has been developed to describe the onset part of the melatonin secretion cycle. The statistical analysis of the results confirmed the consistency of the circadian parameters estimated in both methods. As expected, the melatonin salivary data are highly spread, especially during the night part of the cycle. Due to the observed fluctuations, the criteria for the model quality acceptance were lowered (the percentage of the explained variance (>80%) and the *R* value (>0.89)) compared to the literature data [5, 16, 28].

The fitting method is useful when other methods of the melatonin cycle description are either impossible or impractical [5, 10, 16, 17, 28], in case of difficult (a large statistical spread) or incomplete data, where the bell-shaped model, as the more demanding, cannot be applied [5]. Unfortunately, due to the simplicity of the logit function, it does not allow to estimate DLMOoff or to indicate the location of the release amplitude and calculate the duration of the night melatonin release [4].

The individual differences in the sleep/wake schedules can be also analyzed and described in terms of the patient's chronotype. Because the circadian clocks vary with the sex, age, the genetic background [29], and light exposure [30], the Morningness-Eveningness Chronotype Questionnaires, such as the Munich ChronoType Questionnaire (MCTQ) [31], the Morningness-Eveningness Questionnaire (MEQ) [32], or the Morningness-Eveningness Scale for Children (MESC) [33], may be applied as a simplified estimate of the circadian timing. Importantly, the chronotypes assessed with them are generally strongly correlated with DLMO [34], both in adults [35] and in healthy school-aged children and adolescents [36–38], but the reports on infants are scarce [39, 40]. According to Simpkin et al. and Randler et al. [39, 40], in the toddler age, there is a prevalence of the morning types, but during the next years of age, a progressive delay in chronotype takes place [39], and finally, each type of chronotype can be seen in the preschool children [36, 39, 41]. The differences between the morning and evening chronotypes are seen as a shift of the DLMO values towards the night hours [35]. The morning-type individuals have earlier sleep-wake schedules, earlier diurnal peaks of alertness and performance, and earlier sleep propensity rhythms than the evening-type individuals [42]. On the other hand, an accumulating evidence suggests that there is a feedback between the epileptic seizures and the circadian rhythms—in its consequence, the seizure timing influences the timing of the daily activities, sleeping, and wakefulness, i.e., the chronotype [4, 16, 43] showed that the phase shift of the melatonin release occurs later in the epileptic patients and found that there is a significant relationship between a phase shift of the melatonin peak and the seizures. However, the supporting studies with the application of the chronotype questionnaires were not performed in that study. Our current results confirm the previous observations, as in the HIE children, the DLMO50 and DLMO25 values are shifted to the late night hours too. In accordance with the previous findings, also in this study, in 6 patients (66.6% of the studied group), the epileptic seizures occurred on the day of the melatonin sampling, leading the melatonin secretion in children with hypoxic ischemic encephalopathy to be strongly disturbed. Thus, the obtained results point towards the supposition that, in the HIE children, the evening-type chronotype may dominate. However, the studied group is too small (9 subjects), and the salivary measurements were not supported by the chronotype questionnaire analyses, leaving such supposition uncertain. Because the reports concerning the influence of antiepileptic drugs on the melatonin levels in saliva and plasma of pediatric patients are ambiguous [44–46], we decided not to exclude the patients due to the treatment applied. Along with the small group size, it is the main drawback of the study, but as shown in Tables 2–4, no statistically significant differences between the biofluids and the methods were found. A larger study, based upon the mathematical modelling of the whole melatonin profiles and with application of the chronotype questionnaires, would be necessary to gain a better insight into the disturbances of the circadian rhythms in HIE patients and of their chronotype.

The main disadvantage of the saliva sampling is the lack of the standardized sampling protocols and the standardized normative values enabling the comparison of the results in the tested groups, especially in young children or in epileptic children. In our study, the saliva and blood samples were taken every 1 hour which, according to Crowley et al., allows to estimate the DLMO as accurately as in case for a sampling time of 0.5 hour [47].

Abeysuriya et al. indicate that development of modelling will open new possibilities to calculate and compare the melatonin secretion profiles independently of the biomaterial being tested [26]. Such modelling may allow to establish the normative values for melatonin. We hope that our comparative two-model mathematical approach to evaluation of the melatonin secretion parameters (DLMO) in two biofluids brings us closer to such solution and underlines the role of mathematical modelling.

Generally, higher sampling rate and more data streams used for fitting are necessary to obtain more accurate prospective predictions.

## 5. Conclusions

In this study, we compared the basic parameters of melatonin secretion calculated using the curve-fitting method and the popular threshold method (with a linear interpolation). We showed that the results do not differ statistically, which, in our opinion, argues in favor of using a simple and well-known method being more resistant to imperfect sampling.

Moreover, we compared the results of the determined time parameters (DLMO25 and DLMO50) with those obtained in the blood melatonin measurements from our previous work [5]. Despite the differences in the nature of these biofluids and the sampling schemes (regular blood measurements vs. frequent incomplete data in saliva), we showed that the results of the examined parameters do not differ statistically. In both studies, the different mathematical models were used, but the obtained DLMO parameters agree and do not differ statistically, which allows us to conclude that they could be used interchangeably as needed.

## Figures and Tables

**Table 1:** Patients' characteristics: age 65–80 months and mean age 6 years.

Family history	Nonremarkable, *N* = 3 (33.3%) Hypothyroidism, *N* = 2 (22.2%) Developmental delay, *N* = 2 (22.2%) Epilepsy, *N* = 1 (11.1%) Attention deficit hyperactivity disorder, *N* = 1 (11.1%)

Gestation and delivery period (abnormalities)	Preterm delivery, *N* = 9 (100%) Cesarean section, *N* = 9 (100%) Low birth weight, *N* = 9 (100%)

Psychomotor development	Retarded, *N* = 9 (100%) Mild intellectual disability, *N* = 4 (44.4%) Moderate intellectual disability, *N* = 3 (33.3%) Severe intellectual disability, *N* = 2 (22.2%)

Neurological examination	Spastic diplegia, *N* = 3 (33.3%) Right hemiparesis, *N* = 2 (22.2%) Left hemiparesis, *N* = 2 (22.2%) Spastic tetraplegia, *N* = 2 (22.2%)

Electroencephalography	Generalized paroxysmal changes, *N* = 9 (100%)

Seizures' morphology	Myoclonic seizures (MS), *N* = 9 (100%) Tonic-clonic seizures (GTCS), *N* = 9 (100%)

Antiepileptic treatment	Valproic acid (VPA), *N* = 9 (100%) Levetiracetam (LEV), *N* = 9 (100%)

Brain MRI	Periventricular leukomalacia (PVL), *N* = 9 (100%)

**Table 2:** Comparison of the curve-fitting and threshold-based (with a linear interpolation) methods—the DLMO50, DLMO25, and maximum and minimum melatonin concentrations.

	Average for the curve-fitting method	Average for the threshold-based method	*p* value^*∗∗*^
DLMO_50_ (h)^*∗*^	22.0206	21.9499	0.9437
DLMO_25_ (h)^*∗*^	20.6897	20.8979	0.8415
Maximum melatonin concentration (pg/mL)	63.7445	67.5920	0.7816

^*∗*^Time in the decimal scale; ^*∗∗*^Student's *t*-test.

**Table 3:** Comparison of the curve-fitting and threshold-based (with a linear interpolation) methods—the minimum melatonin concentrations.

	Median for curve-fitting method	Median for threshold-based method	*p* value^*∗*^
Minimum melatonin concentration (pg/mL)	2.6433	0.7800	0.7239

^*∗*^Mann–Whitney *U* test.

**Table 4:** The comparison of the DLMO parameters obtained from the salivary and blood melatonin secretion profiles.

	Median for the saliva samples	Median for the blood samples	*p* value^*∗∗*^
DLMO50 (h)^*∗*^	22.29	22.85^*∗∗∗*^	0.2510
DLMO25 (h)^*∗*^	21.19	21.90^*∗∗∗*^	0.2893

^*∗*^Time in the decimal scale. ^*∗∗*^Mann–Whitney *U* test. ^*∗∗∗*^Data from Paprocka et al., 2018.

## Data Availability

The datasets generated for this study are available from the corresponding author upon request.
